# Supplementary figures and images for: Efficient training approaches for optimizing behavioral performance and reducing head fixation time
Source: PLoS One. 2022 Nov 10;17(11):e0276531. doi: 10.1371/journal.pone.0276531 (PMC9648727; doi:10.1371/journal.pone.0276531)

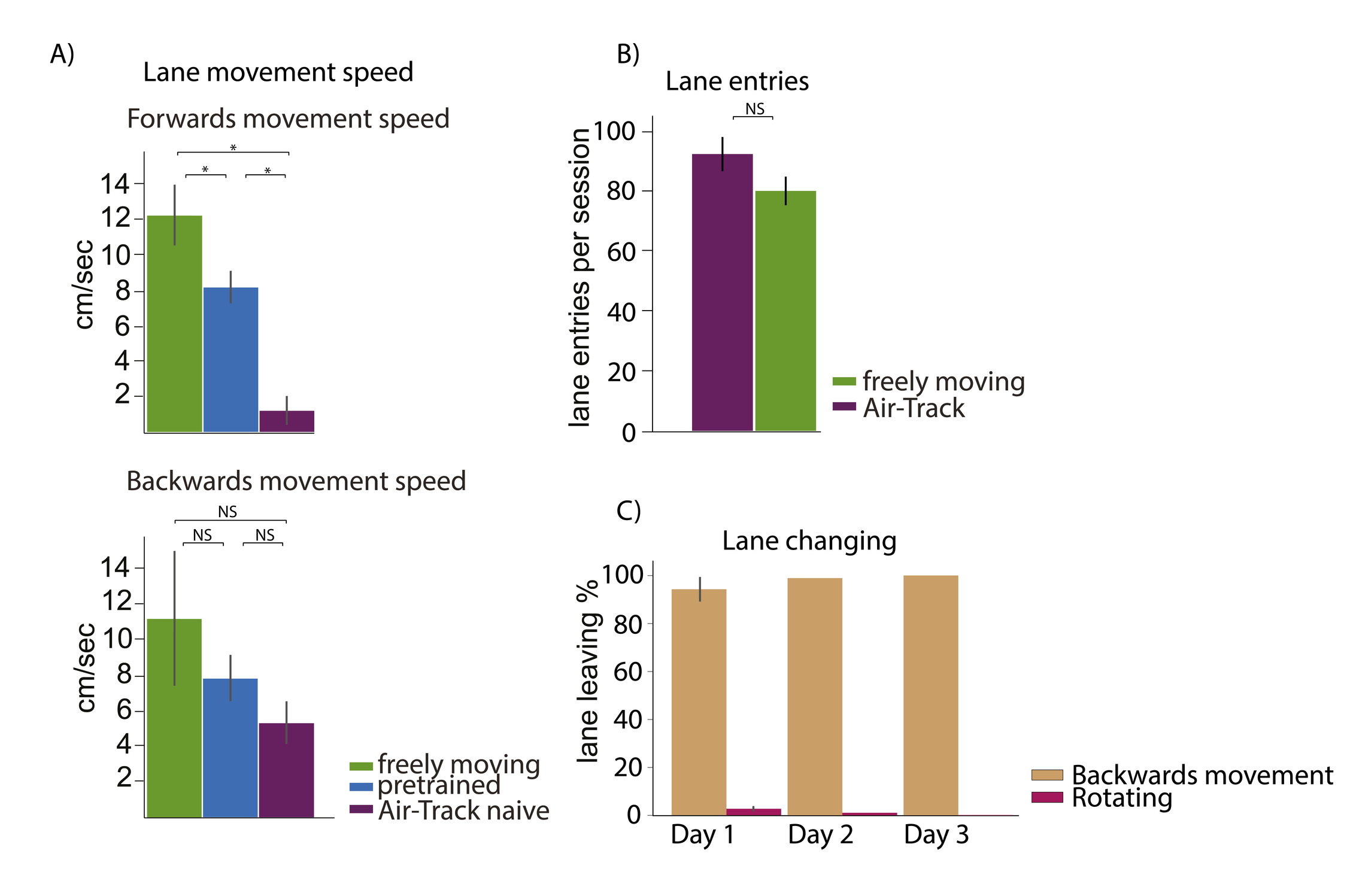

Supplement: S1 Fig — A) Comparing the movement speed of freely moving mice with naive head fixed and pre-trained head fixed mice.* p<0.05, NS p>0.05 via a two tailed T test B) Comparing the lane entries for head fixed and home-cage mice, showed no significance p>0.05 C) Freely moving mice in the home-cage are able to rotate in the lane, but prefer to move backwards out of the lane. With no turning around at day 3. (TIF) [file pone.0276531.s001.tif]

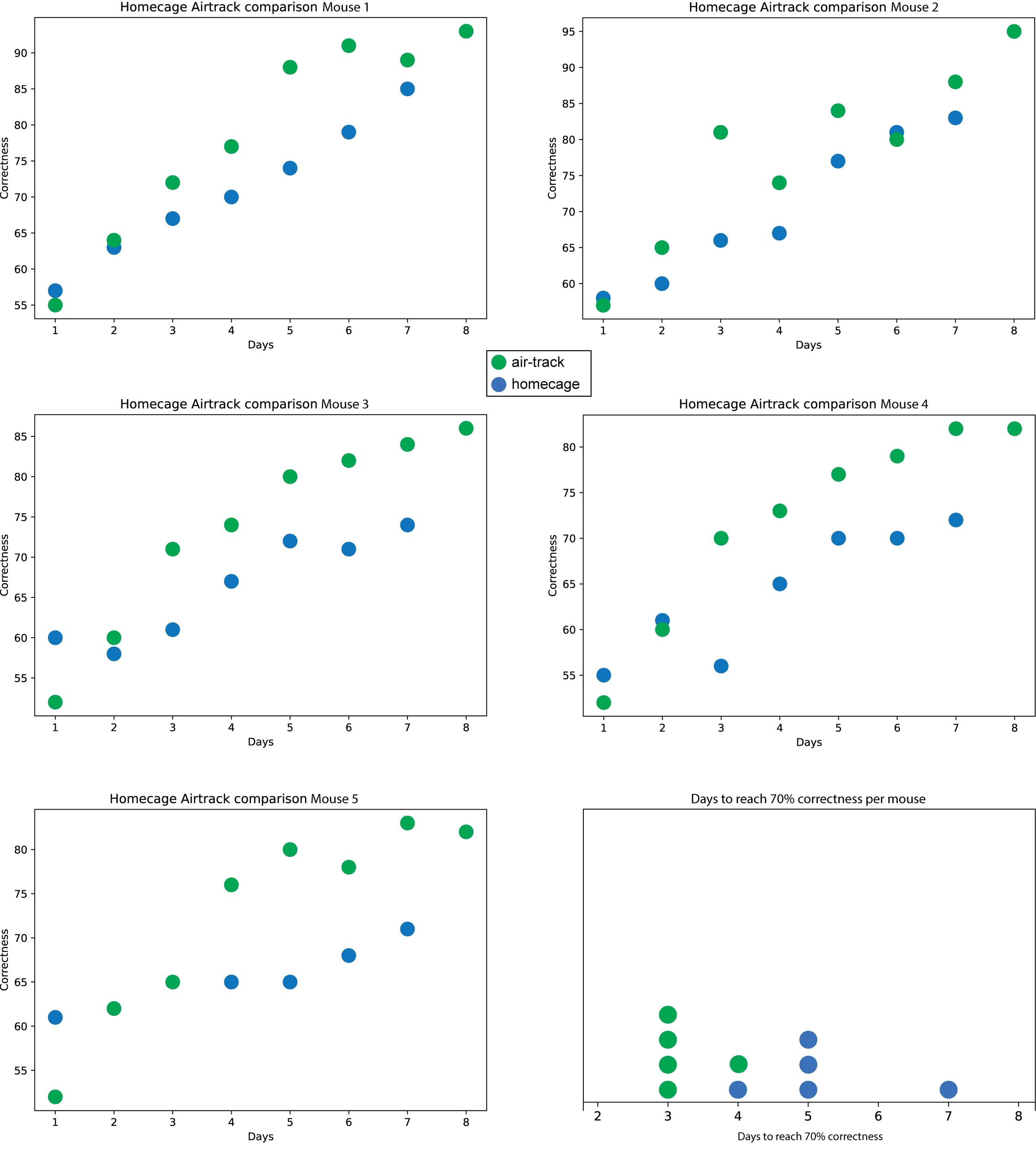

Supplement: S2 Fig — Each dot plot represents a single mouse which was trained in the home-cage as well as the Air-Track, as well as a line plot showing the time point each mouse reaches the 70% correctness. (TIF) [file pone.0276531.s002.tif]

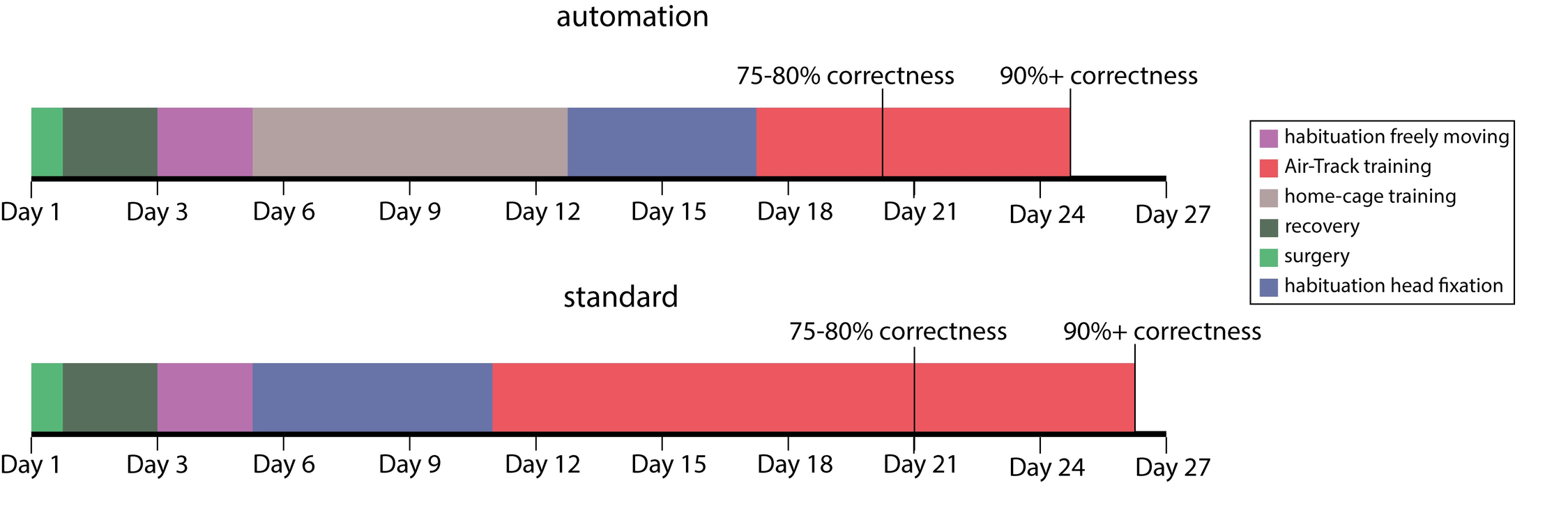

Supplement: S3 Fig — Representation of the average time in days it takes to train a single mouse, including surgery, recovery, and training in the traditional standard way of training mice for the Air-Track and the automated home-cage training. (TIF) [file pone.0276531.s003.tif]
