# Supplementary material for: Efficient training approaches for optimizing behavioral performance and reducing head fixation time
Source: PLoS One. 2022 Nov 10;17(11):e0276531. doi: 10.1371/journal.pone.0276531 (PMC9648727; doi:10.1371/journal.pone.0276531)
Supplement: Attachment — Submitted filename: Response to Reviewers.pdf [file pone.0276531.s004.pdf]

Dear Sir / Madam,
Thank you for your reviews.
Since our lab prefers to use Google Docs as a tool to change and update, the paper is the marked-up copy written and updated with Google Docs and therefore saved as docx format. The finished manuscript is written in LaTeX, following the style and formatting guidelines of PLOS ONE. In addition to the changes requested by the reviewers, we made some additional minor changes to improve readability. We updated Figure 3 and Figure 5. In Figure 3 we switched the label of Part B and C. Figure 5 was changed according to the request of the reviewer.

1. Please ensure that your manuscript meets PLOS ONE's style requirements, including those for file naming. The PLOS ONE style templates can be found at https://journals.plos.org/plosone/s/file?id=wjVg/PLOSOne_formatting_sample_main_body.pdf and https://journals.plos.org/plosone/s/file?id=ba62/PLOSOne_formatting_sample_title_authors_affiliations.pdf

We followed the guidelines

2. Please update your submission to use the PLOS LaTeX template. The template and more information on our requirements for LaTeX submissions can be found at http://journals.plos.org/plosone/s/latex.

We used the template

3. We note that the grant information you provided in the 'Funding Information' and 'Financial Disclosure' sections do not match. When you resubmit, please ensure that you provide the correct grant numbers for the awards you received for your study in the 'Funding Information' section.

We will correct the mistake.

4. Thank you for stating the following financial disclosure:

The following funding sources have supported this project: (1) Deutsche Forschungsgemeinschaft (DFG), Grant Nos. 246731133, 250048060 and 267823436 to ML; (2) DFG Project number 327654276 – SFB 1315 to ML; (3) European Commission Horizon 2020 Research And Innovation Program and Euratom Research and Training Program 2014–2018 (under grant agreement No. 670118 to ML); (4) Human Brain Project, EU Commission Grant 720270 (SGA1), 785907 (SGA2) and 945539 (SGA3) to ML; (5) Einstein Foundation Berlin EVF-2017-363 to ML.

Please state what role the funders took in the study. If the funders had no role, please state: "The funders had no role in study design, data collection and analysis, decision to publish, or preparation of the manuscript."

If this statement is not correct you must amend it as needed.

Please include this amended Role of Funder statement in your cover letter; we will change the online submission form on your behalf.

The funders had no role in study design, data collection and analysis, decision to publish, or preparation of the manuscript

5. We note that you have stated that you will provide repository information for your data at acceptance. Should your manuscript be accepted for publication, we will hold it until you provide the relevant accession numbers or DOIs necessary to access your data. If you wish to make changes to your Data Availability statement, please describe these changes in your cover letter and we will update your Data Availability statement to reflect the information you provide.

The repository we have provided is the following:

https://gin.g-node.org/nasra/Prior-experience-accelerates-training-of-head-fixed-mice-on-a-floating-platform.git

We will provide the relevant data in this repository once the manuscript has been accepted for publication.

6. Please amend your list of authors on the manuscript to ensure that each author is linked to an affiliation. Authors' affiliations should reflect the institution where the work was done (if authors moved subsequently, you can also list the new affiliation stating "current affiliation:…." as necessary).'

We have updated the list of authors.

Reviewers' comments:

Reviewer's Responses to Questions

Comments to the Author

1. Is the manuscript technically sound, and do the data support the conclusions?

The manuscript must describe a technically sound piece of scientific research with data that supports the conclusions. Experiments must have been conducted rigorously, with appropriate controls, replication, and sample sizes. The conclusions must be drawn appropriately based on the data presented.

Reviewer #1: Yes

Reviewer #2: Yes

---

2. Has the statistical analysis been performed appropriately and rigorously?

Reviewer #1: Yes

Reviewer #2: Yes

---

3. Have the authors made all data underlying the findings in their manuscript fully available?

The [PLOS Data policy] requires authors to make all data underlying the findings described in their manuscript fully available without restriction, with rare exception (please refer to the Data Availability Statement in the manuscript PDF file). The data should be provided as part of the manuscript or its supporting information, or deposited to a public repository. For example, in addition to summary statistics, the data points behind means, medians and variance measures should be available. If there are restrictions on publicly sharing data—e.g. participant privacy or use of data from a third party—those must be specified.

Reviewer #1: Yes

Reviewer #2: No

https://gin.g-node.org/nasra/Prior-experience-accelerates-training-of-head-fixed-mice-on-a-floating-platform.git

---

4. Is the manuscript presented in an intelligible fashion and written in standard English?

PLOS ONE does not copy edit accepted manuscripts, so the language in submitted articles must be clear, correct, and unambiguous. Any typographical or grammatical errors should be corrected at revision, so please note any specific errors here.

Reviewer #1: Yes

Reviewer #2: Yes

---

5. Review Comments to the Author

Please use the space provided to explain your answers to the questions above. You may also include additional comments for the author, including concerns about dual publication, research ethics, or publication ethics. (Please upload your review as an attachment if it exceeds 20,000 characters)

Reviewer #1: The authors in this paper investigate how pretraining with freely moving animals can be used to improve the learning rates in head-immobilized tasks. Specifically, the authors utilize a unique two-choice discrimination task with a Y maze that can be used with both freely moving and head-immobilized animals. They use this apparatus to compare the learning rates of three different groups of mice: a) Ones in the freely moving task b) Naïve ones while head-fixed and c) head-fixed animals that have been pre-trained in the freely moving task. The authors find a significant decrease in training required for the last group of animals.

The question addressed in this paper is of great importance to the field. Given the requirement of many advanced recording techniques for head-immobilized animals together with complex behavior, their results provide support for using pre-training with complex behavioral tasks that the animals can generalize while head-posted. Moreover, their Y-maze design is easy to generalize over and can also be useful for many behavioral questions in the field.

Even though the authors try to address a relevant question with a good behavioral task that can be applied in both freely moving and head-immobilized animals, I feel like it needs a bit more work to become much more useful to the field.

Major:

1. Quantification and comparison of the behavioral patterns between the freely moving and head-posted version of the task is lacking a bit. Given the very short length of the results section the authors could augment them by looking into different parts of the behavior for already collected data. For example:

a. As the authors have used the same animals between the two conditions, it would be interesting to know how the animal variability looks in such a task. For example, the authors could investigate the difference between the performance criterion for the two tasks across animals (each axis represents the performance criterion for the two tasks and for each animal in a scatter plot).

We thank the reviewer for this suggestion and have prepared plots for each mouse, in Supplementary Figure 2.

b. Animals can perform complex movements in both tasks, but only the movement speed is compared. It would be easy to compare other aspects of the movement such as distance traveled, rotations or even # of lane entries.

We agree and have generated some comparative figures (Supplementary Figure 2). One comparison that we have not made is of distance traveled. Mice in the home-cage can leave the training area and move around freely, so the travel distance is not comparable to the limited environment of the Air-Track.

c. It would be important to know the maximum performance that these animals can reach in each version of the task. Do mice that are trained on the air platform without pretraining reach similar levels of performance to pretrained animals?

Thank you for the suggestion, we added more details to Figure 5 showing the final performance of the naïve Air-Track mice. This figure shows the total time it takes for mice to reach the same level as the pre-trained mice.

2. Why is the performance of the freely moving task lower than the head-posted task? Is it the extra training the animals received, or is it because of the "animals being more focused" as they mention in the discussion? In the discussion, the authors need to clearly mention this. Is it also possible that animals need more days under water restriction to reach stable motivation levels, as has been shown in rats (Vasilev et al. 2021)?

The pre-trained mice transfer their knowledge of the freely moving task to the Air-Track, which results in a continuation of the learning curve. The drop in performance in the first 3 days is due to the fact that even after being habituated to head fixation, and mice still need to learn to move the Air-Track and need to be motivated to perform the task.

3. In Figure 6A I probably miss something. For the home cage training to reach criterion the animals required about 7 days of training which is >10hours per animal. In the bar plot of Fig. 6A the home-cage training takes <1hour. Why is that?

We have rewritten and clarified Figure 6 legend. ***Figure 6. Active time investment to reach criterion levels**. A) The total time in hours the researcher spends training mice is less due to the home cage training (left bar). The biggest effect of home-cage training is the reduction in time taken during learning of the task in the Airtrack. Habituation to the freely moving environment and home-cage training are unique to automation. Since those two phases are done in the automated home-cage it requires only a few minutes per day to check on the mice and the rest is done automatically. B) The time saving aspect in relation to home cage training. C) Comparison of the total time investment in hours. As the number of mice being trained increases, there is a substantial effect of home-cage training on time needed to train mice.*

4. Is the backwards movement defined as a reference to the platform or the animals? From the discussion, you mention freely moving animals moving backwards under certain circumstances, but I am surprised that animals can move 10cm/sec backwards. If is relative to the platform (so animals rotate while head-posted and then move backwards) would make sense.

The movement speed was calculated by using a basler high speed camera and the software ZRView. This allowed us to count the frames between the end of the lane and the center of the Y maze. Using this method, we obtained the number of frames it takes to move from the end of the lane back to the center. The number of frames divided by the frame rate resulted in a cm/sec value.

5. I don't think that the authors provide evidence that "most-natural behaviors are unaffected", as I find this is too general of a statement. Maybe the performance in this task is unaffected, but I would suggest the authors tone down this statement. A nice experiment that would offer support for such a claim would be to train animals on the air-platform and then see if it improves the performance in a freely moving task. I don't know if the camera used had enough resolution, but it would be also interesting to check for snout /whisker movements as they have been shown to correlate with sampling of peripersonal space (Kurnikova et al. 2017). Even though that might be beyond the scope of this work, it would offer support for this head-immobilized task being a "natural behavior". Do you expect to have such behaviors or to have them disappear, like the disconjugate eye movements?

We agree with the reviewer, and have rewritten the paragraph. "**We conclude that home-cage pre-training improves learning performance of head fixed mice and that while head fixation obviously limits some aspects of movement, the patterns of behavior observed in head fixed and freely moving mice are similar.**"

Our cameras did not have the resolution to track whiskers, and were not in a position to track eye movement. We do expect that in the course of behavior, in freely moving animals, eye movement would be disconjugate, and both whisking and eye movement would be linked to head movement. We have tracked both eyes and whiskers in mice head fixed in a plus maze and show that eye movement is conjugate and related to whisker movement(Bergmann et., al 2021). They concluded that not only do head-fixed mice move their eyes, but saccadic eye movement is coordinated with the asymmetric positioning of the whiskers.

Minor:

1. A schematic representation of the various timing of training events would be very helpful, since this information is not in one place in the text and not very easy to find.

Thank you for pointing this out. We have fixed this and added a supplementary figure (Supplementary Figure 3).

2. Why is there a different criterion applied in figures 5 and 6 (70% vs 75%)?

Thank you for noticing this was a typo, and the criterion is now uniformly 70%.

3. Text edit: "The mice were acclimated to being handled, and held by the head post during which they obtained a reward when the head post was manually held in place by the experimenter" would be better as: "The mice were acclimated to being handled, during which the animals obtained a reward while the head post was manually held in place by the experimenter"

We have rewritten this sentence: "The mice were acclimated to being handled, during which the animals obtained a reward while the head post was manually held in place by the experimenter."

4. I would replace the "improve the learning curve" with "improve the learning rate".

We implemented this change: "The fundamental issue which we tried to address here was to improve the learning rate of mice and in the course of doing so we also examined how and whether head fixation changed behavior. Our work suggests that pre-training animals under freely-moving conditions reduced the total time the animals spent learning while head fixed and that head fixation does not trigger aberrant behavior.   "

5. "To minimize the time between home-cage training and Air-Track training as well as outside factors did all the mice undergo the surgery at the beginning of the experiment. " -> "… outside factors, all mice underwent surgery …"

We have rewritten this sentence.:" To minimize the time between home-cage training and Air-Track training as well as outside factors, all mice underwent surgery at the beginning of the experiment."

6. Two paragraphs after "Daily training routine: Common to both home-cage and head fixed training" are repeated.

Thank you for pointing this out, we removed the duplicate paragraph.

7. Figure 3 should be used in the bias describing paragraph, not 4.

We have corrected this error.

8. "To answer the question regarding the naturality of the head fixed behavior, were the same mice, first used in the home-cage system and then transferred to the Air-Track" Sentence not clear.

We have rewritten this paragraph: To assess whether home-cage training significantly changed the rate of learning, we measured performance (percentage correct) and the average speed of movement. To address whether head fixation fundamentally alters mouse behavior, we used the same mice first in the home-cage system then in the Air-Track. This approach makes it possible to assess the effect of prior training on learning the behavior in the Air-track and provides an opportunity to assess the effect of head fixation on behavior mice in the Y-maze when freely moving, and when head fixed in the Air-track.

References:

Kurnikova et al. Coordination of Orofacial Motor Actions into Exploratory Behavior by Rat. Current Biology 2017, 27 (5), 688–696.

Vasilev, D. et al. Three Water Restriction Schedules Used in Rodent Behavioral Tasks Transiently Impair Growth and Differentially Evoke a Stress Hormone Response without Causing Dehydration. eNeuro 8, (2021).

Reviewer #2: The author's compare training of mice in either head fixed or freely moving task and show that pre-training for headfixed tasks has some benefit, furthermore performance is not greatly compromised by headfixation. This work is important for any labs developing high-throughput means of assessment.

"This is in contrast to the earlier work where mice had access to water for 7 hrs each day, and over 3 weeks of training mice only achieved 55% success rates in a cue-based licking task

(Murphy 2020)." Perhaps it was not clear but in Murphy et al. 2020 mice were trained both in a 7 h a day mode and 24/7 (44 mice 24/7) and 8 females 7 h a day with similar performance.

We have rewritten the entire paragraph:Training mice in their home-cage has been a central goal of earlier work of the lab of Murphy and colleagues (2018; 2020). While their work did not address whether mice learned faster when trained while freely moving, their work showed that mice could be trained to learn all aspects of behavior in their home-cages. The design of their home cage included several additional features that we did not deploy, including self-head fixation and imaging neural activity in the home-cage after mice self-head fix. One additional difference between their task design and ours, was that in our design, mice could perform the task and have access to water rewards for only 1 hour each day. In the earlier work from Murphy, they used two groups, in the first group mice could perform24/7 hrs and over 3 weeks of training mice achieved 55% success rates and the second smaller group had access for 7hrs per day with 35% success rate in a cue-based licking task (Murphy 2020). In another study, where mice could perform the task at any time, mice reached criterion levels within 31-37 days (Tejapratap BolluGoldberg et al., 2018). In our version of the home cage training, mice had access to water for an hour each day, and in this hour they had to perform the task. Limiting the task to an hour, and limiting the availability of water reward to a single hour, focused mice on learning and performing the task. In our initial experiments (not shown) where mice had 24/7 access to the training area for 3 days and could perform the task at any time, mice showed no evidence that they were learning the task. When we changed the design, mice learned the task and performance reached criterion within 7 days of training.

Need to state that pixycam only works with visible light (colored).

We have rewritten this sentence to: When a mouse entered the maze, the identity of the mouse was checked via the color tracking algorithm and Pixy camera (see below) deployed to track colors (under visible light) painted on the head post of each animal.

Gallinares et al should be Galiñanes et al. and is NOT in the reference list

We have corrected this error.

Other references are out of order or missing Murphy et al. 2017

The reference order has been fixed, and the missing references have been added.

please carefully check the manscript and references for accuracy

Thank you very much for pointing it out. We went over all references and corrected the inaccuracies as well as added more references.

https://gin.g-node.org/nasra/Prior-experience-accelerates-training-of-head-fixed-miceon-

a-floating-platform

Please excuse the inconvenience. The link should work now.

https://gin.g-node.org/nasra/Prior-experience-accelerates-training-of-head-fixed-mice-on-a-floating-platform.git

---

6. PLOS authors have the option to publish the peer review history of their article (what does this mean?). If published, this will include your full peer review and any attached files.

If you choose “no”, your identity will remain anonymous but your review may still be made public.

Do you want your identity to be public for this peer review? For information about this choice, including consent withdrawal, please see our Privacy Policy.

Reviewer #1: No

Reviewer #2: No
