# Supplementary material for: Efficient training approaches for optimizing behavioral performance and reducing head fixation time
Source: PLoS One. 2022 Nov 10;17(11):e0276531. doi: 10.1371/journal.pone.0276531 (PMC9648727; doi:10.1371/journal.pone.0276531)
Supplement: Attachment — Submitted filename: Response to review.pdf [file pone.0276531.s005.pdf]

To the editor and reviewers.

The manuscript marked-up copy was written and updated using Google Docs and then saved as docx format. The finished manuscript is written in LaTeX, following the style and formatting guidelines of PLOS ONE. In addition to the changes requested by the reviewers, we made some additional minor changes to improve readability.

Reviewer #1: I would like to thank the authors for addressing all of my concerns.

I only have few minor points:

Fig.3e, Fig4, Fig5, Supp. Fig.1a, would benefit if the statistical tests and the p values are mentioned in the figure legends.

We added the p values in the description for Fig.3e, Fig4, Fig5, Supp. Fig.1a

Supp Fig1c : Is the "rotating" bar from Day3 missing? Also are some of the error bars missing?

Thank you for your comment. Supp Fig 1 C was in the original draft for the publication, but we removed it, since we did not see a good way to present the data. For day 2 there was no visible error bar because the standard deviation is less than 1%, but we added a minimal error bar for visual representation and for Day 3 there is no bar due to the fact that not a single mouse rotated in the maze after the second day. But we added a highlight at 0 for visualization.

The response to my point 1a is not exactly what I had in mind (days to criterion for each task in the two axis) but the data in Supp. Fig. 2 do provide the relevant information. For visibility purposes, the authors can make that a line plot.

We have added the line plot.

Reviewer #2: no further issues important, nice to have the new citations about training this seems like a good paper they might cite this

We added the citation:

Vasilev, D. et al. Three Water Restriction Schedules Used in Rodent Behavioral Tasks Transiently Impair Growth and Differentially Evoke a Stress Hormone Response without Causing Dehydration. eNeuro 8, (2021)
